# Procrastination and risky health behaviors: a possible way to nurture health promotion among young adults in Italy

**DOI:** 10.3389/fpubh.2024.1432763

**Published:** 2024-08-22

**Authors:** Francesca Licata, Emma Antonia Citrino, Riccardo Maruca, Gianfranco Di Gennaro, Aida Bianco

**Affiliations:** ^1^Department of Health Sciences, School of Medicine, University of Catanzaro "Magna Græcia", Catanzaro, Italy; ^2^Department of Medical and Surgical Sciences, School of Medicine University of Catanzaro "Magna Græcia", Catanzaro, Italy

**Keywords:** health promotion, Italy, procrastination, risky behaviors, university students

## Abstract

**Background:**

The study’s main objectives were to evaluate the distribution of levels of procrastination and its relationship with sleep quality, smoking status, alcohol consumption, and skipping breakfast, as a proxy measure of an unhealthy dietary pattern, among Italian university students.

**Methods:**

A cross-sectional study was conducted at the “Magna Græcia” University of Catanzaro in the Southern part of Italy, using stratified random sampling techniques. Eligible students were sent an anonymous online survey aimed at assessing sociodemographic characteristics, procrastination levels using the Pure Procrastination Scale, sleep quality using Pittsburgh Sleep Quality Index, smoking status, alcohol consumption using WHO’s Alcohol Use Disorders Identification Tool, and breakfast habits.

**Results:**

The study included 518 participants with a mean age of 23 year. More than half of the sample was enrolled in medicine or life science majors and the procrastination mean score was 15 (±5.9 SD). Being procrastinators was significantly more frequently among students who were poor sleepers, hazardous alcohol consumers and breakfast skippers. When analyzing the clustering of risky behaviors, it was found that as the number of risky behaviors increased, the procrastination score exhibited an exponential increase.

**Conclusion:**

The study findings showed that university students who engage in procrastination tend to adopt risky health behaviors. The data gathered could be useful to derive targeted interventions aimed at groups more exposed to harmful health behaviors and to encourage institutional policies to promote healthy lifestyles within universities. Universities can act as hubs for cultivating a culture of well-being and promoting a healthy environment.

## Introduction

Procrastination is known as the voluntary delay of the intended course of action, even when one expects that the possible negative effects will outweigh the benefits (1). People procrastinate as a temporary solution to avoid negative feelings associated with a particular task or event.

Although the concept of procrastination may appear straightforward, developing valid methods to quantify procrastination has proven challenging over time. Early research primarily relied on various self-report measures such as the Decisional Procrastination Questionnaire (2), the Academic Procrastination State Inventory (APSI) (3), the General Procrastination Scale (4), the Adult Inventory of Procrastination (5), and the Aitken Procrastination Inventory (6) that attempted to explain procrastination as a general trait. However, findings from a meta-analysis of procrastination’s causes and effects, conducted by Steel (7) suggested that procrastination is better understood as a unidimensional construct. Consequently, the Pure Procrastination Scale (PPS) was introduced as a reliable and comprehensive measure of procrastination, across various contexts (8).

Evidence has accumulated that the harms from procrastination are not limited to those involving productivity at work or academic achievement, but they include detrimental effects on health and well-being, especially when procrastination becomes a chronic behavior (9). The link between procrastination and health was first observed in a longitudinal study showing that students who regularly procrastinated experienced less stress and fewer health issues at the beginning of the term compared to those who did not procrastinate (10). However, the initial benefits of procrastination reversed by the end of the term, increasing stress and exacerbating health problems of procrastinators compared to non-procrastinators. Although the study did not investigate the reasons for this connection, it was suggested that the intense stress associated with chronic procrastination might explain the poorer health outcomes for procrastinators. Based on this foundational study, a theoretical explanation for why chronic procrastination is associated with a greater number of health problems was proposed (11, 12).

Health risk behaviors are a significant issue among university students (13). Commonly, the vast majority of students begin their university studies before the age of 20, staying at the university for 4 or 5 years, constituting a crucial period for the acquisition and consolidation of health behaviors. The course of university is frequently accompanied by new unhealthy habitual behaviors that could impact students’ health and lifestyles into adulthood (14, 15). Lifestyles characterized by adequate nutrition, healthy physical activity, restful sleep, no tobacco smoking, and moderate alcohol consumption help to protect health and reduce the risk of non-communicable diseases (16). Moreover, the university context, where self-organization and time management are key factors, represents a good example of an environment where also tendency to procrastinate can thrive. Indeed, estimates indicate that 70–95% of college students engage in procrastination (7, 17), with approximately 75% considering themselves procrastinators and nearly 50% procrastinating consistently and problematically (18).

There is growing recognition that university students are an important target-population for public health policymaking. The period of university study represents a great opportunity, as universities could promote a healthy study environment. A solid and continuously updated database regarding student health and well-being could be useful to derive targeted and sustainable interventions aimed at groups more exposed to harmful health behaviors. Gathering data in this area is intended to encourage institutional policies to promote healthy lifestyles within universities. To the best of our knowledge, few studies (9, 19–22) have focused on the relationship between procrastination and multiple unhealthy behaviors. Therefore, the study’s main objectives were to evaluate the distribution of levels of procrastination and its relationship with sleep quality, smoking status, alcohol consumption, and skipping breakfast, as a proxy measure of an unhealthy dietary pattern, among Italian university students.

## Materials and methods

### Participant recruitment and sampling

A cross-sectional study was conducted in different majors of “Magna Græcia” University of Catanzaro, in Southern Italy. Data collection took place between the 13th and the 28th of February 2023 using the stratified random sampling technique. Stratification was based on majors (i.e., medicine and life sciences, social sciences, and technology) and the stratifying variable was the year of study. The sampling frame of the students was available for each stratum, and a random sample of students, proportional to size, was selected from each stratum. The sample size was calculated using the level of precision formula. Being under 18 years old and lacking proficiency in the Italian language were listed as exclusion criteria. All the eligible students received an email with a link to an anonymous online survey. Participants were assured that their responses would remain confidential and would only be used for research purposes. The email also emphasized the voluntary nature of participation. Only those who provided their informed consent were taken into consideration for the study.

### Questionnaire design

The survey comprised six sections. Four questions were included in the questionnaire’s initial part (1) to examine social and demographic variables. The PPS was used in the second segment (2) to assess levels of procrastination. The third section (3) of the questionnaire evaluated the sleep quality using the Pittsburgh Sleep Quality Index (PSQI). In the survey’s fourth component (4), examining smoking status, participants were asked if they had ever smoked at least 100 cigarettes and, if so, how often on average they did it (daily, sometimes, or not at all). The fifth section (5) examined unhealthy alcohol consumption, using the screening tool known as the Alcohol Use Disorders Identification Tool (AUDIT). In the last section (6) skipping breakfast and its frequency were investigated as a proxy measure of unhealthy dietary patterns.

### Measures

#### Procrastination

Procrastination, defined as the act of voluntarily delaying an intended course of action despite expecting to be worse off for the delay (8), was measured using the short Italian version of PPS which includes items 4 through 8 from the full PPS. In detail, the 5 items were “In preparation for some deadlines, I often waste time by doing other things,” “Even jobs that require little else except sitting down and doing them, I find that they seldom get done for days,” “I often find myself performing tasks that I had intended to do days before,” “I am continually saying ‘I’ll do it tomorrow’” and “I generally delay before starting on work I have to do.” Responses were rated on a Likert scale from 1 (“very rarely or does not represent me”) to 5 (“very often or always represents me”). The procrastination score varied from 5 to 25 based on the sum of the ratings. The validation of the Italian version of the instrument has been performed by Svartdal et al. (23) through a rigorous process of translation and back translation and cross-cultural validation of the original version. The tool has demonstrated good internal consistency (Cronbach’s α >0.88) and it seems appropriate to be used as a core procrastination measure (23).

#### Sleep quality

The validated Italian version of the PSQI (24) was used to investigate sleep quality among the recruited students. The PSQI is the most commonly self-rated measure used in clinical and research settings and it has shown good validity and reliability in university student samples (25). The instrument evaluates seven clinical domains of sleep difficulties (i.e., subjective sleep quality, sleep latency, sleep duration, habitual sleep efficiency, sleep disturbances, use of sleeping medications, and daytime dysfunction). Of the 19 PSQI items, 15 are rated on a scale of 0–3 and 4 are open-ended and recoded to a scale of 0–3. The higher the score (>5) the lower the sleep quality. The participants were provided instructions on how to complete this part of the questionnaire.

#### Smoking status

The smoking status of the participants was defined according to the classification provided by the Center for Disease Control and Prevention (26). People were categorized as non-smokers (i.e., when they never smoked or smoked less than 100 cigarettes in their lifetime), former smokers (i.e., when they smoked at least 100 cigarettes in their lifetime but had quit at the time of data collection), and current smokers (i.e., those who smoked 100 cigarettes in their lifetime and who currently smoke cigarettes, both occasionally and daily) (26). Participants who reported not knowing if they had smoked or not at least 100 cigarettes in their lifetime were classified as undefined.

#### Alcohol consumption

The WHO’s AUDIT was used to investigate alcohol use (27). This 10-item assessment is considered an accurate tool for identifying university students who use alcohol in hazardous ways. It measures the three domains of alcohol consumption, such as intake (items 1–3), dependence (items 4–6), and adverse consequences of drinking (items 7–10) (27). The Italian-translated version of the AUDIT is currently accepted and used within the Italian context. Eight questions have a five-point rating system that goes from 0 to 4, while questions nine and 10 have a three-point rating system that goes from 0 to 4. The final score might range between 0 and 40 points. Hazardous alcohol consumption is defined as having a threshold of 8 or above, which increases the risk of negative consequences for the user and endangers the public’s health.

#### Skipping breakfast

The item “How many days a week do you eat breakfast?” was utilized to investigate the breakfast skipping habit. The response options varied from 0 to 7 days a week, and those who skipped breakfast more than three times per week were defined as breakfast skippers (28). The choice to adopt skipping breakfast as a proxy measure of unhealthy dietary patterns aimed to capture a broader spectrum of dietary habits that may influence health outcomes. It was supported by some studies showing that skipping breakfast is associated with lower academic performance, poorer mental health, and an increased likelihood of engaging in other risky health behaviors (29, 30).

### Statistical analysis

All the variables were summarized by mean and standard deviation when normally distributed, and by median and interquartile range (IQR) when skewness was present. Data distribution was investigated by Shapiro–Wilk test. Categorical variables were expressed as counts and percentages.

The level of procrastination among subjects was calculated by summing all procrastination-related items in the questionnaire. Univariate analyses were conducted to explore the relationship between procrastination score and sociodemographic characteristics, poor sleep habits, alcohol consumption, smoking habits, and breakfast skipping. *T*-tests were performed to determine if there was a significant difference in means if samples were normally distributed; the Wilcoxon-Mann–Whitney test was used if normality was violated. The association of procrastination score with poor sleep habits, alcohol consumption, smoking habits, and breakfast skipping, was analyzed using multiple linear regression (Model 1). To evaluate how the aggregation of unhealthy habits is associated with the level of procrastination, an additional model including the clustering of risky behaviors altogether (as a count from 0 to 4) was built (Model 2). In both linear regression models, estimates were adjusted by including age, gender, and university majors attended, as possible confounders. Moreover, to explore possible predictors of the relationship between procrastination and risky behaviours, the interaction terms of sociodemographic characteristics (i.e., age, gender, and the major attended) with each of the unhealthy behavior (i.e., poor sleep quality, hazardous alcohol consumption, smoking, and breakfast skipping) and with the clustering of unhealthy behaviors were tested. The linear model was checked for assumptions of linearity (augmented partial residual plot), homoscedasticity (Breusch-Pagan test), normality of residuals (kernel density plot), and the presence of influential observations (studentized residuals investigation). Statistical analysis was developed using Stata Statistical Software, Version 18 (31).

### Ethical consideration

This study received the approval of the Local Human Research Ethics Committee (ID 36/2023/10/18) and it was conducted in accordance with the Helsinki Declaration.

## Results

The descriptive characteristics of the study participants are shown in Table 1. The study included 518 participants (74.7% of women) with a mean age of 23 years (±3.1 SD). More than half of the sample (59.6%) was enrolled in medicine or life science majors. The procrastination mean score was 15 (±5.9 SD). For women, the mean procrastination score was 14.2 (±5.8 SD), while men exhibited a slightly higher mean score of 15.2 (±6.2 SD). However, the difference was not statistically significant (*p* = 0.555). Examining academic majors, students in medicine or life sciences reported a mean procrastination score of 14.5 (±5.9 SD); in contrast, those who attended majors in social sciences and technology had a higher mean score of 15.7 (±5.9 SD). The divergence in procrastination scores was statistically significant (*p* = 0.034).

**Table 1:** Distribution of Pure Procrastination Scale total score according to general characteristics of the study population.

	Participants, No. (%)		
	Full sample	Pure Procrastination Scale total score	
Characteristics	(*N* = 518)	Mean	SD
Gender			
Female	387 (74.7)	14.2	±5.8
Male	131 (25.3)	15.2	±6.2
		*p*-value: 0.555	
Majors			
Medical or life sciences	309 (59.6)	14.5	±5.9
Social sciences or Technology	209 (40.4)	15.7	±5.9
		*p*-value: 0.034	
Sleep quality			
Good sleepers	220 (42.5)	13.6	±5.8
Poor sleepers	298 (57.5)	16.1	±5.8
		*p*-value: <0.001	
Smoking status*			
Non-smokers	309 (65.9)	14.7	±5.8
Current smokers	160 (34.1)	15.5	±6.0
		*p*-value: 0.205	
Alcohol consumption			
No/Non-hazardous alcohol consumers	469 (90.5)	14.7	±5.8
Hazardous alcohol consumers**	49 (9.5)	17.9	±5.9
		*p*-value: <0.001	
Breakfast skipping			
Breakfast eaters	398 (76.8)	14.5	±5.6
Breakfast skippers	120 (23.2)	16.7	±6.3
		*p*-value: <0.001	

*Those who responded: “I do not know” to the question “Have you smoked 100 or more cigarettes in your life?” along with former smokers were excluded.

**AUDIT score ≥ 8.

### Sleep quality

Examining sleep quality those reporting good sleep quality exhibited a mean procrastination score of 13.6 (±5.8 SD), while individuals experiencing poor sleep quality demonstrated a notably higher mean score of 16.1 (±5.8 SD). This discrepancy was statistically significant, with individuals having poor sleep quality showing increasing procrastination scores (*p* < 0.001).

### Smoking habits

Our investigation into smoking habits showed non-smokers exhibiting a mean procrastination score of 14.7 (±5.8 SD), whereas smokers exhibited a higher mean score of 15.5 (±6 SD). The difference in procrastination scores was not statistically significant (*p* = 0.205).

### Alcohol consumption

Analyzing alcohol consumption patterns demonstrated noteworthy insights. Individuals engaging in non-hazardous alcohol consumption displayed a mean procrastination score of 14.7 (±5.8 SD), while those with hazardous alcohol consumption exhibited a substantially higher mean score of 17.9 (±5.9 SD). The distinction in procrastination scores was statistically significant (*p* < 0.001).

### Skipping breakfast

Finally, individuals who reported skipping breakfast exhibited a mean procrastination score of 16.7 (±6.3 SD), whereas those who consumed breakfast showed a lower mean score of 14.5 (±5.6 SD). The difference in procrastination scores was statistically significant (*p* < 0.001).

### Linear regression model results regarding the procrastination-health relationship

The results of the linear regression model investigating the relationship between procrastination and each of the selected risky behavior showed that the procrastination score was significantly higher among students who were poor sleepers (coeff. 2.27, 95%CI 1.21–3.33), hazardous alcohol consumers (coeff. 2.25, 95%CI 0.46–4.04) and breakfast skippers (coeff. 1.68, 95%CI 0.42–2.95) compared to their counterparts (Model 1 in Table 2). The results of the models testing the effect of the interaction on predicting the procrastination score are reported in Supplementary Table 1. The joint effect of being woman and breakfast skipper resulted in almost a 3-point decrease (coeff. −2.96, 95%CI−5.62 – −0.30) of the procrastination score compared to the male counterpart. No significant interaction on the procrastination score was shown when testing sociodemographic characteristics and being poor sleepers (age: coeff. 0.07, 95%CI−0.27 – 0.42; female gender: coeff. 0.25, 95%CI−2.12 – 2.63; attending medicine or life sciences majors: coeff. −0.62, 95%CI−2.74 – 1.50), current smokers (age: coeff−0.23, 95%CI−0.59 – 0.12; female gender: coeff. −0.23, 95%CI−2.67 – 2.21; attending medicine or life sciences majors: coeff. 0.49, 95%CI−1.71 − 2.68), hazardous alcohol consumers (age: coeff. 0.32, 95%CI−0.31 – 0.94; female gender: coeff. −2.92, 95%CI−6.44 – 0.60; attending medicine or life sciences majors: coeff. 2.01, 95%CI−1.45 – 5.48), and breakfast skippers (age: coeff. 0.19, 95%CI−0.20 – 0.57; attending medicine or life sciences majors: coeff. −2.14, 95%CI−4.63 − 0.35).

**Table 2:** Results of the linear regression models for potential predictors of High Pure Procrastination Score.

Model 1. *F* (7,461) = 6.31, *p* < 0.0001, *R*^2^ = 8.7%, adjusted *R*^2^ = 7.4%; Obs = 469				
Variables	Coefficient	95% CI		*p*-value
Age in years, continous	0.13	−0.04	0.30	0.120
Gender				
Male*	1.00			
Female	−0.77	−1.97	0.42	0.202
Majors attended				
Social sciences or Technology*	1.00			
Medical or Life Science	−0.91	−1.96	0.15	0.091
Sleep quality				
Good sleepers*	1.00			
Poor sleepers	2.27	1.21	3.33	<0.001
Smoking status				
Non-smokers*	1.00			
Current smokers	−0.14	−1.27	0.99	0.811
Alcohol consumption				
No/Non-hazardous alcohol consumers *	1.00			
Hazardous alcohol consumers	2.25	0.46	4.04	0.014
Breakfast skipping				
Breakfast eaters*	1.00			
Breakfast skippers	1.68	0.42	2.95	0.009

Model 2. *F* (6,462) = 5.65, *p* < 0.0001, *R*^2^ = 6.8%, adjusted *R*^2^ = 5.6%; Obs = 469				
Variables	Coefficient	95% CI		*p*-value
Age in years, continous	0.14	−0.03	0.31	0.116
Gender				
Male*	1.00			
Female	−0.62	−1.81	0.57	0.307
Majors attended				
Social sciences or Technology*	1.00			
Medical or Life Science	−0.95	−2.01	0.11	0.079
Number of unhealthy behaviors				
0*	1.00			
1	2.00	0.76	3.23	<0.001
2	2.17	0.67	3.67	0.005
3	6.27	3.68	8.85	<0.001

*Reference category.

When analyzing the clustering of risky behaviors (Model 2 in Table 2), it was found that as the number of risky behaviors increased, the procrastination score exhibited an exponential increase, ranging from a 2-point increase (95%CI 0.76–3.23) among students with only one behavior to 2.17 (95%CI 0.67–3.67) and 6.27 (95%CI 3.68–8.85) in subjects with 2 and 3 risky behaviors, respectively. Furthermore, no significant interaction was found in all the models testing the effect on the procrastination score of the students’ sociodemographic characteristics and having reported one unhealthy behavior (age: coeff. 0.11, 95%CI−0.29 – 0.52, female gender: coeff. −0.76, 95%CI−3.54 – 2.03; and attending medicine or life sciences majors: coeff. −0.45, 95%CI−2.98 – 2.07); two unhealthy behaviors (age: coeff. −0.12, 95%CI−0.62 – 0.38; female gender: coeff. 1.07, 95%CI−2.35 – 4.48; and attending medicine or life sciences majors: coeff. 0.37, 95%CI−2.65 – 3.40) and three unhealthy behaviors (age: coeff. 0.55, 95%CI−0.46 – 1.56; female gender: coeff. −3.23, 95%CI−8.46 – 1.99; and attending medicine or life sciences majors: coeff. 1.30, 95%CI−3.98 – 6.58) compared to having not reported unhealthy behaviors (Supplementary Table 2).

## Discussion

From a public health perspective, understanding the intricate interplay between procrastination and risky health behaviors holds paramount significance. Many of the healthy behaviors effective for the prevention of diseases are difficult to adopt into daily life and may encourage procrastination (11). Bad habits are hard to break. The reason people that know why and how to live a healthy lifestyle fail to do so could be related with the fact that the reward of a healthy lifestyle is a delayed gratification whereas most of less healthy choices often offer instant gratification, and most people have problems with deferring the gratification. The current study provides novel insights that may be useful for tailoring on-campus health promotion activities to target university student segments.

At first glance, the results shed light on the relationship between procrastination and unhealthy behaviors. Specifically, we found that higher levels of procrastination were associated with an increased likelihood of engaging in poor sleep habits, alcohol abuse, and unhealthy eating patterns. This finding is consistent with prior research that links chronic procrastination with unhealthy behaviors (32). The statistically significant difference in procrastination scores between those classified as poor sleepers and their peers with better sleep quality demonstrated by our analysis corroborates that sleep is an important factor to consider while evaluating procrastination (33, 34). It could be argued that because sleep has been strongly correlated to self-regulation skills (35) and that poor sleep may affect one’s capacity for self-regulation (36), perceived bad sleep quality may correlate with procrastination levels in our sample.

Similarly to those classified as bad sleepers, students engaging in alcohol abuse displayed higher procrastination scores, indicating a noteworthy correlation between these behaviors. University students worldwide drink more alcohol than non-university students (37). Drinking alcohol is linked to high levels of stress because it is thought to relieve tension (38–40). Nevertheless, drinking too much alcohol has negative effects on one’s physical and mental well-being, which makes it harder to function socially, interpersonally, and academically. Therefore, this vicious cycle of procrastination and alcohol consumption might worsen stress levels and result in poor academic performance.

Furthermore, students who reported skipping breakfast also exhibited higher levels of procrastination, suggesting a potential link between unhealthy eating habits and procrastination tendency. Although some people find it easy to stick with intermittent fasting, time-restricted eating is a largely debated topic nowadays. Indeed, evidence has accumulated that breakfast skipping is frequently associated with poor health outcomes (29, 30), and it is especially true when taking into consideration the university student population (41). In addition, evidence suggests that the metabolic conditions for food intake are optimal in the morning (42, 43). It is well known that breakfast helps university students to function better cognitively and psychosocially. Hence, the impact of nutrition on mood and energy levels can directly affect a student’s motivation and ability to focus on tasks, leading to increased procrastination.

Procrastinators often find themselves overwhelmed by all the steps involved in even the most simple tasks (e.g., having breakfast) and therefore fail to take any action. It could be pivotal in helping individuals develop the ability to regulate or manage their emotions, identify their needs, prioritize tasks, make difficult decisions, and, more importantly, initiate healthy actions.

An intriguing discovery of the study emerged when the risky behavior composite score was scrutinized. Participants engaging in more than one risky health behavior exhibited double or even triple the likelihood of being categorized as procrastinators. On the one hand, our results underscore the interconnected nature of risky behaviors and suggest that procrastination may have broader implications for students, impacting their decision-making abilities and overall well-being. On the other hand, the results highlight the importance of addressing procrastination as a multifaceted issue that goes beyond simply managing time and deadlines.

In the authors opinion implementing procrastination interventions within the setting of students’ everyday lives (i.e., university context) may be a cost-effective strategy to promote health-protective behaviors, given the high prevalence of students citing procrastination as a significant problem. Addressing the context within which individuals live can increase the likelihood of success because it offers opportunities to situate practice in their own context. To create awareness among universities of these many facets and how they can impede academic success might effectively help students who struggle with procrastination, and given the demonstrated connection between procrastination and unhealthy behaviors help students protect their own health. Unhealthy behaviors are modifiable risk factors contributing to the global burden of non-communicable diseases (16). Therefore, universities can act as hubs for cultivating a culture of well-being and increasing the required awareness to change any deeply ingrained behavior pattern. Hence, leveraging universities as centers for fostering well-being not only benefits individual students but also has far-reaching implications for public health. Moreover, universities could face the students’ health creating an exhaustive set of programs to address the different needs of each student in terms of well-being. For instance, universities might provide free consultations by working with medical experts and actively promoting them on campus. Students may grow inside and out of the classroom by having direct access to nutrition counseling, exercise programs, and stress management workshops. Furthermore, sharing information about these services via campus events and social media channels can raise student awareness and utilization even more.

This study has some limitations that have to be acknowledged to appreciate its findings. The first limitation of the study design is that the findings could be viewed as scantly informative for causal inference. Nevertheless, the findings provide valuable information on procrastination and risky health behaviors that should not be dismissed. Indeed, information from cross-sectional studies is pivotal in providing baseline data to generate hypotheses for further research. Second, the behaviors were self-reported and only a proxy measure of real behaviors, introducing the risk of social desirability bias. This bias was limited by assuring participants that their responses would be anonymous and could not be traced back to them. Furthermore, procrastination was measured through a self-assessed questionnaire (i.e., PPS), and someone could argue that it could not correspond with objective measures. However, PPS showed good psychometric properties and produced a reliable assessment of procrastination. Self-report instruments have several advantages, such as being easy to fill out, and data being available almost immediately, whereas observing procrastination in real-life situations would require more resources and time.

Even though the cross-sectional design prevents a causal conclusion, the study emphasizes the link between procrastination and unhealthy behaviors, including poor sleep habits, alcohol abuse, and unhealthy dietary patterns among university students. A setting-based approach that integrates actions across different risk factors, including procrastination, could maximize health promotion and curb the burden of non-communicable diseases.

## Data Availability

The original data presented in the study are openly available in Mendeley Data repository at https://doi.org/10.17632/s9bgnzgbkf.1.
