# Supplementary material for: Procrastination and risky health behaviors: a possible way to nurture health promotion among young adults in Italy
Source: Front Public Health. 2024 Aug 22;12:1432763. doi: 10.3389/fpubh.2024.1432763 (PMC11374597; doi:10.3389/fpubh.2024.1432763)
Supplement: Supplementary file 1 [file Table_1.docx]

| **Table 1. Results of the linear regression models** **to explore the interaction terms of sociodemographic characteristics of the participants with each of the selected unhealthy behaviors on the Pure Procrastination Scale (PPS) total score (obs. no. 469)** | | | | | | | | | | | | | | | | | | | | | | | | | | |
| --- | --- | --- | --- | --- | --- | --- | --- | --- | --- | --- | --- | --- | --- | --- | --- | --- | --- | --- | --- | --- | --- | --- | --- | --- | --- | --- |
|  | **Model 1^#^** | | **Model 2** | | **Model 3** | | **Model 4** | | **Model 5** | | **Model 6** | | **Model 7** | | **Model 8** | | **Model 9** | | **Model 10** | | **Model 11** | | **Model 12** | | **Model 13** | |
|  | *F (7,461) = 6.31, p<0.0001, R2=8.7%, adjusted R2=7.4%* | | *F (8,460) = 5.53, p<0.0001, R2=8.7%, adjusted R2=7.2%* | | *F (8,460)=5.74, p<0.0001, R2=9.1%, adjusted R2=7.5%* | | *F (8,460)=5.64, p<0.0001, R2=8.9%, adjusted R2=7.4%* | | *F (8,460)=5.63, p<0.0001, R2=8.9%, adjusted R2=7.3%* | | *F (8,460)=5.51, p<0.0001, R2=8.8%, adjusted R2=7.2%* | | *F (8,460)=5.51, p<0.0001, R2=8.7%, adjusted R2=7.2%* | | *F (8,460)=5.87, p<0.0001, R2=9.3%, adjusted R2=7.7%* | | *F (8,460) = 6.16, p< 0.0001, R2 = 9.7%, adjusted R2 = 8.1%* | | *F (8,460)=5.55, p<0.0001, R2=8.8%, adjusted R2=7.2%* | | *F (8,460)=5.53, p<0.0001, R2=8.8%, adjusted R2=7.2%* | | *F (8,460)=5.68, p<0.0001, R2=9%, adjusted R2=7.4%* | | *F (8,460)=5.90, p<0.0001, R2=9.3%, adjusted R2=7.7%* | |
| **Variables** | **Coeff.** | **95%CI** | **Coeff.** | **95%CI** | **Coeff.** | **95%CI** | **Coeff.** | **95%CI** | **Coeff.** | **95%CI** | **Coeff.** | **95%CI** | **Coeff.** | **95%CI** | **Coeff.** | **95%CI** | **Coeff.** | **95%CI** | **Coeff.** | **95%CI** | **Coeff.** | **95%CI** | **Coeff.** | **95%CI** | **Coeff.** | **95%CI** |
| **Age in years, continuous** | 0.13 | -0.04, 0.3 | 0.09 | -0.18, 0.36 | 0.21 | 0.01, 0.42 | 0.11 | -0.67, -0.29 | 0.89 | -0.11, -0.28 | 0.13 | -0.34, -0.30 | -0.13 | -0.36, -0.30 | 0.13 | -0.36, -0.30 | 0.13 | -0.04, 0.30 | 0.14 | -0.33, 0.31 | 0.14 | -0.03, 0.31 | 0.14 | -0.03, 0.31 | 0.14 | -0.03, 0.31 |
| **Gender** |  |  |  |  |  |  |  |  |  |  |  |  |  |  |  |  |  |  |  |  |  |  |  |  |  |  |
| Male* | 1.00 |  | 1.00 |  | 1.00 |  | 1.00 |  | 1.00 |  | 1.00 |  | 1.00 |  | 1.00 |  | 1.00 |  | 1.00 |  | 1.00 |  | 1.00 |  | 1.00 |  |
| Female | -0.77 | -1.9, 0.42 | -0.76 | -1.96, 0.43 | -0.83 | -2.03, 0.36 | -0.75 | -1.94, -0.44 | -0.75 | -1.94, -0.44 | -0.90 | -2.57, 0.77 | -0.69 | -2.18, -0.80 | -0.39 | -1.67, -0.88 | 0.01 | -1.37, 1.39 | -0.78 | -1.98, -0.41 | -0.76 | -1.95, 0.44 | -0.74 | -1.93, 0.46 | -0.80 | -1.99, 0.39 |
| **Majors attended** |  |  |  |  |  |  |  |  |  |  |  |  |  |  |  |  |  |  |  |  |  |  |  |  |  |  |
| Social sciences or Technology* | 1.00 |  | 1.00 |  | 1.00 |  | 1.00 |  | 1.00 |  | 1.00 |  | 1.00 |  | 1.00 |  | 1.00 |  | 1.00 |  | 1.00 |  | 1.00 |  | 1.00 |  |
| Medical or Life Science | -0.91 | -1.96, 0.15 | -0.91 | -1.97, 0.14 | -0.93 | -1.98, 0.13 | -0.88 | -1.94, -0.17 | -0.92 | -1.98, -0.13 | -0.90 | -1.96, 0.15 | 2.27 | 1.21, 3.33 | -0.95 | -2.00, -0.11 | -0.94 | -1.99, 0.11 | -0.55 | -2.17, 1.07 | -1.07 | -2.37, 0.22 | -1.11 | -2.23, -0.00 | -0.42 | -1.62, 0.77 |
| **Sleep quality** |  |  |  |  |  |  |  |  |  |  |  |  |  |  |  |  |  |  |  |  |  |  |  |  |  |  |
| Good sleepers* | 1.00 |  | 1.00 |  | 1.00 |  | 1.00 |  | 1.00 |  | 1.00 |  | 1.00 |  | 1.00 |  | 1.00 |  | 1.00 |  | 1.00 |  | 1.00 |  | 1.00 |  |
| Poor sleepers | 2.27 | 1.21, 3.33 | 0.61 | -7.27, 8.50 | 2.28 | 1.22, 3.34 | 2.29 | 1.23, 3.35 | 2.26 | 1.20, 3.31 | 2.09 | -0.04, 4.13 | 0.03 | -2.09, 2.14 | 2.21 | 1.15, 3.27 | 2.17 | 1.11, 3.23 | 2.64 | 1.00, 4.27 | 2.28 | 1.22, 3.34 | 2.23 | 1.14, 3.29 | 2.31 | 1.25, 3.36 |
| **Smoking status** |  |  |  |  |  |  |  |  |  |  |  |  |  |  |  |  |  |  |  |  |  |  |  |  |  |  |
| Non-smokers***** | 1.00 |  | 1.00 |  | 1.00 |  | 1.00 |  | 1.00 |  | 1.00 |  | 1.00 |  | 1.00 |  | 1.00 |  | 1.00 |  | 1.00 |  | 1.00 |  | 1.00 |  |
| Current smokers | -0.14 | -1.27, 0.99 | -0.14 | -1.28, 1.00 | -0.23 | -0.59, -0.12 | -0.12 | -1.26, 1.01 | -0.17 | -1.30, -0.97 | -0.14 | -1.28, 1.00 | 0.03 | -2.09, 2.14 | -0.20 | -1.33, 0.94 | -0.11 | -1.24, 1.02 | -0.16 | -1.30, 0.98 | -0.42 | -2.12, 1.28 | -0.16 | -1.30, 0.97 | -0.15 | -1.29, 0.98 |
| **Alcohol consumption** |  |  |  |  |  |  |  |  |  |  |  |  |  |  |  |  |  |  |  |  |  |  |  |  |  |  |
| No/Non-hazardous alcohol consumers * | 1.00 |  | 1.00 |  | 1.00 |  | 1.00 |  | 1.00 |  | 1.00 |  | 1.00 |  | 1.00 |  | 1.00 |  | 1.00 |  | 1.00 |  | 1.00 |  | 1.00 |  |
| Hazardous alcohol consumers | 2.25 | 0.46, 4.04 | 2.26 | -0.47, 4.06 | 9.38 | 4.54, 14.22 | -4.80 | -18.91, 9.32 | 2.24 | -0.45, 4.03 | 2.26 | -0.46, 4.06 | 2.22 | -0.41, 4.04 | 4.00 | 1.23, 6.76 | 2.23 | -0.44, 4.01 | 2.28 | 0.49, 4.08 | 2.24 | 0.45, 4.04 | 1.29 | -1.15, 3.72 | 2.39 | 0.6, 4.19 |
| **Breakfast skipping** |  |  |  |  |  |  |  |  |  |  |  |  |  |  |  |  |  |  |  |  |  |  |  |  |  |  |
| Breakfast eaters* | 1.00 |  | 1.00 |  | 1.00 |  | 1.00 |  | 1.00 |  | 1.00 |  | 1.00 |  | 1.00 |  | 1.00 |  | 1.00 |  | 1.00 |  | 1.00 |  | 1.00 |  |
| Breakfast skippers | 1.68 | 0.42, 2.95 | 1.68 | 0.41, 2.94 | 1.72 | -0.45, 2.98 | 1.66 | -0.39, 2.93 | -2.53 | -11.29, 6.22 | 1.69 | -0.42, 2.96 | 1.69 | 0.42, 2.96 | 1.74 | 0.48, 3.01 | 3.78 | 1.52, 6.04 | 1.70 | 0.43, 2.97 | 1.68 | 0.41, 2.95 | 1.59 | 0.32, 2.87 | 2.98 | 1.01, 4.95 |
| **Unhealthy behaviours with age** |  |  |  |  |  |  |  |  |  |  |  |  |  |  |  |  |  |  |  |  |  |  |  |  |  |  |
| **Sleep quality** |  |  |  |  |  |  |  |  |  |  |  |  |  |  |  |  |  |  |  |  |  |  |  |  |  |  |
| Good sleepers* | - |  | 1.00 |  | - |  | - |  | - |  | - |  | - |  | - |  | - |  | - |  | - |  | - |  | - |  |
| Poor sleepers | - |  | 0.07 | -0.27, 042 | - |  | - |  | - |  | - |  | - |  | - |  | - |  | - |  | - |  | - |  | - |  |
| **Smoking status** |  |  |  |  |  |  |  |  |  |  |  |  |  |  |  |  |  |  |  |  |  |  |  |  |  |  |
| Non-smokers* | - |  | - |  | 1.00 |  | - |  | - |  | - |  | - |  | - |  | - |  | - |  | - |  | - |  | - |  |
| Current smokers | - |  | - |  | -0.23 | -0.59, 0.12 | - |  | - |  | - |  | - |  | - |  | - |  | - |  | - |  | - |  | - |  |
| **Alcohol consumption** |  |  |  |  |  |  |  |  |  |  |  |  |  |  |  |  |  |  |  |  |  |  |  |  |  |  |
| No/Non-hazardous alcohol consumers * | - |  | - |  | - |  | 1.00 |  | - |  | - |  | - |  | - |  | - |  | - |  | - |  | - |  | - |  |
| Hazardous alcohol consumers | - |  | - |  | - |  | 0.31 | -0.31, -0.94 | - |  | - |  | - |  | - |  | - |  | - |  | - |  | - |  | - |  |
| **Breakfast skipping** |  |  |  |  |  |  |  |  |  |  |  |  |  |  |  |  |  |  |  |  |  |  |  |  |  |  |
| Breakfast eaters* | - |  | - |  | - |  | - |  | 1.00 |  | - |  | - |  | - |  | - |  | - |  | - |  | - |  | - |  |
| Breakfast skippers | - |  | - |  | - |  | - |  | -0.19 | -0.11, -0.28 | - |  | - |  | - |  | - |  | - |  | - |  | - |  | - |  |
| **Unhealthy behaviours with female gender** |  |  |  |  |  |  |  |  |  |  |  |  |  |  |  |  |  |  |  |  |  |  |  |  |  |  |
| **Sleep quality** |  |  |  |  |  |  |  |  |  |  |  |  |  |  |  |  |  |  |  |  |  |  |  |  |  |  |
| Good sleepers* | - |  | - |  | - |  | - |  | - |  | 1.00 |  | - |  | - |  | - |  | - |  | - |  | - |  | - |  |
| Poor sleepers | - |  | - |  | - |  | - |  | - |  | 0.25 | -2.12, 2.63 | - |  | - |  | - |  | - |  | - |  | - |  | - |  |
| **Smoking status** |  |  |  |  |  |  |  |  |  |  |  |  |  |  |  |  |  |  |  |  |  |  |  |  |  |  |
| Non-smokers* | - |  | - |  | - |  | - |  | - |  | - |  | 1.00 |  | - |  | - |  | - |  | - |  | - |  | - |  |
| Current smokers | - |  | - |  | - |  | - |  | - |  | - |  | -0.23 | -2.67, 2.21 | - |  | - |  | - |  | - |  | - |  | - |  |
| **Alcohol consumption** |  |  |  |  |  |  |  |  |  |  |  |  |  |  |  |  |  |  |  |  |  |  |  |  |  |  |
| No/Non-hazardous alcohol consumers * | - |  | - |  | - |  | - |  | - |  | - |  | - |  | 1.00 |  | - |  | - |  | - |  | - |  | - |  |
| Hazardous alcohol consumers | - |  | - |  | - |  | - |  | - |  | - |  | - |  | -2.92 | -6.44, 0.60 | - |  | - |  | - |  | - |  | - |  |
| **Breakfast skipping** |  |  |  |  |  |  |  |  |  |  |  |  |  |  |  |  |  |  |  |  |  |  |  |  |  |  |
| Breakfast eaters* | - |  | - |  | - |  | - |  | - |  | - |  | - |  | - |  | 1.00 |  | - |  | - |  | - |  | - |  |
| Breakfast skippers | - |  | - |  | - |  | - |  | - |  | - |  | - |  | - |  | -2.96 | -5.62, -0.30 | - |  | - |  | - |  | - |  |
| **Unhealthy behaviours with attending medical or life science majors** |  |  |  |  |  |  |  |  |  |  |  |  |  |  |  |  |  |  |  |  |  |  |  |  |  |  |
| **Sleep quality** |  |  |  |  |  |  |  |  |  |  |  |  |  |  |  |  |  |  |  |  |  |  |  |  |  |  |
| Good sleepers* | - |  | - |  | - |  | - |  | - |  | - |  | - |  | - |  | - |  | 1.00 |  | - |  | - |  | - |  |
| Poor sleepers | - |  | - |  | - |  | - |  | - |  | - |  | - |  | - |  | - |  | -0.62 | -2.74, 1.50 | - |  | - |  | - |  |
| **Smoking status** |  |  |  |  |  |  |  |  |  |  |  |  |  |  |  |  |  |  |  |  |  |  |  |  |  |  |
| Non-smokers* | - |  | - |  | - |  | - |  | - |  | - |  | - |  | - |  | - |  | - |  | 1.00 |  | - |  | - |  |
| Current smokers | - |  | - |  | - |  | - |  | - |  | - |  | - |  | - |  | - |  | - |  | 0.49 | -1.71, 2.68 | - |  | - |  |
| **Alcohol consumption** |  |  |  |  |  |  |  |  |  |  |  |  |  |  |  |  |  |  |  |  |  |  |  |  |  |  |
| No/Non-hazardous alcohol consumers * | - |  | - |  | - |  | - |  | - |  | - |  | - |  | - |  | - |  | - |  | - |  | 1.00 |  | - |  |
| Hazardous alcohol consumers | - |  | - |  | - |  | - |  | - |  | - |  | - |  | - |  | - |  | - |  | - |  | 2.01 | -1.45, 5.48 | - |  |
| **Breakfast skipping** |  |  |  |  |  |  |  |  |  |  |  |  |  |  |  |  |  |  |  |  |  |  |  |  |  |  |
| Breakfast eaters* | - |  | - |  | - |  | - |  | - |  | - |  | - |  | - |  | - |  | - |  | - |  | - |  | 1.00 |  |
| Breakfast skippers | - |  | - |  | - |  | - |  | - |  | - |  | - |  | - |  | - |  | - |  | - |  | - |  | -2.14 | -4.63, 0.35 |
| *Reference category  **^#^**Model presented in the Table 2 of the study as Model 1 | | | | | | | | | | | | | | | | | | | | | | | | | | |
