# Supplementary material for: Procrastination and risky health behaviors: a possible way to nurture health promotion among young adults in Italy
Source: Front Public Health. 2024 Aug 22;12:1432763. doi: 10.3389/fpubh.2024.1432763 (PMC11374597; doi:10.3389/fpubh.2024.1432763)
Supplement: Supplementary file 2 [file Table_2.docx]

| **Table 2. Results of the linear logistic regression models** **to explore the interaction terms of sociodemographic characteristics of the participants with the number of unhealthy behaviors on the Pure Procrastination Scale (PPS) total score (obs. no. 469)** | | | | | | | | |
| --- | --- | --- | --- | --- | --- | --- | --- | --- |
|  | **Model 1^#^** | | **Model 2** | | **Model 3** | | **Model 4** | |
|  | *F(6,462)= 5.65, p<0.0001, R2=6.8%, adjusted R2= 5.6%* | | *F (9,459)= 4.01, p=0.0001, R2=7.3%, adjusted R2= 5.5%* | | *F(9,459)= 4.07, p<0.0001, R2=7.4%, adjusted R2= 5.6%* | | *F(9,459)= 3.83, p=0.0001, R2=7%, adjusted R2= 5.2%* | |
| **Variables** | **Coeff.** | **95%CI** | **Coeff.** | **95%CI** | **Coeff.** | **95%CI** | **Coeff.** | **95%CI** |
| **Age in years, continuous** | 0.14 | -0.03, 0.31 | 0.09 | -0.25, 0.42 | -0.32 | -2.40, 1.77 | 0.14 | -0.03, 0.31 |
| **Gender** |  |  |  |  |  |  |  |  |
| Male* | 1.00 |  | 1.00 |  | 1.00 |  | 1.00 |  |
| Female | -0.62 | -1.81, 0.57 | -0.67 | -1.87, 0.53 | 0.15 | -0.02, 0.32 | -0.59 | -1.79, 0.62 |
| **Majors attended** |  |  |  |  |  |  |  |  |
| Social sciences or Technology* | 1.00 |  | 1.00 |  | 1.00 |  | 1.00 |  |
| Medical or Life Science | -0.95 | -2.01, 0.11 | -0.94 | -2.00, 0.12 | -1.02 | -2.08, 0.05 | -0.88 | -2.88, 1.12 |
| **Number of unhealthy behaviors** |  |  |  |  |  |  |  |  |
| 0* | 1.00 |  | 1.00 |  | 1.00 |  | 1.00 |  |
| 1 | 2.00 | 0.76, 3.23 | -0.58 | -9.95, 8.79 | 2.55 | 0.18, 4.93 | 2.26 | 0.30, 4.23 |
| 2 | 2.17 | 0.67, 3.67 | 4.98 | -6.64, 16.60 | 1.33 | -1.60, 4.25 | 1.99 | -0.27, 4.25 |
| 3 | 6.27 | 3.68, 8.85 | -5.93 | -28.48, 16.61 | 7.95 | 4.15, 11.74 | 5.50 | 1.46, 9.55 |
| **Number of unhealthy behaviors with age** |  |  |  |  |  |  |  |  |
| 0* | - |  | 1.00 |  | - |  | - |  |
| 1 | - |  | 0.11 | -0.29,0.52 | - |  | - |  |
| 2 | - |  | -0.12 | -0.62, 0.38 | - |  | - |  |
| 3 | - |  | 0.55 | -0.46, 1.56 | - |  | - |  |
| **Number of unhealthy with female gender** |  |  |  |  |  |  |  |  |
| 0* | - |  | - |  | 1.00 |  | - |  |
| 1 | - |  | - |  | -0.76 | -3.54, 2.03 | - |  |
| 2 | - |  | - |  | 1.07 | -2.34, 4.48 | - |  |
| 3 | - |  | - |  | -3.23 | -8.46, 1.99 | - |  |
| **Number of unhealthy with attending medical or life sciences majors** |  |  |  |  |  |  |  |  |
| 0* | - |  | - |  | - |  | 1.00 |  |
| 1 | - |  | - |  | - |  | -0.45 | -2.98, 2.07 |
| 2 | - |  | - |  | - |  | 0.37 | -2.65, 3.40 |
| 3 | - |  | - |  | - |  | 1.30 | -3.98, 6.58 |
| *Reference category  **^#^**Model presented in the Table 2 of the study as Model 2 | | | | | | | | |
